# Supplementary material for: Facial expression recognition using visible and IR by early fusion of deep learning with attention mechanism
Source: PeerJ Comput Sci. 2025 Mar 12;11:e2676. doi: 10.7717/peerj-cs.2676 (PMC11935750; doi:10.7717/peerj-cs.2676)
Supplement: Supplemental Information 2 [file peerj-cs-11-2676-s002.docx]

**Supplementary File**

**5.3. Evaluation Indicators**

Multiple metrics are available for evaluating our proposed models. In this study, we use evaluation metrics derived from the confusion matrix, including Accuracy, Precision, Recall, and F1-score as shown in Eqs. (1)–(4). Supplementary-Table 1 shows the confusion matrix.

 **Accuracy**: The ratio of correctly predicted samples to the total number of samples.

 **Precision**: The ratio of true positive predictions to the total predicted positives.

 **Recall**: The ratio of true positive predictions to the total actual positives.

 **F1-Score**: The harmonic mean of precision and recall.

$$Accuracy=\frac{TP+TN}{TP+TN+FP+FN} (1)$$

$$Precision=\frac{TP}{TP+FP} (2)$$

$$Recall=\frac{TP}{TP+FN} (3)$$

$$F1-Score=\frac{2\times TP}{2\times TP+FP+FN} (4)$$

**Supplementary-Fig. 1 and Supplementary-Fig. 2** illustrate the performance of the ResNet-18 model over 50 epochs on the visible dataset from the VIRI Database, comparing results with and without the **CosineAnnealingLR scheduler**.

In **Supplementary-Fig. 1** (A), where the **CosineAnnealingLR scheduler** is applied, the training accuracy (green line, A) rapidly approaches near-perfect levels, stabilizing close to 1.0, indicating that the model has effectively learned the features of the training data. The validation accuracy (blue line, A), however, fluctuates between 0.65 and 0.85, showing some instability. This suggests that while the model generalizes reasonably well, it could benefit from further tuning or regularization to reduce overfitting, as indicated by the gap between training and validation accuracy. On the **Supplementary-Fig. 1(B)** loss curve, the training loss remains relatively low and stable, while the validation loss shows minor oscillations without a clear downward trend. This may indicate that the model is converging but has reached a plateau in generalization capability, due to limitations in the dataset.

On the other hand, in **Supplementary-Fig. 2** (B), after removing the **CosineAnnealingLR scheduler**, the accuracy and loss curves show similar patterns. The training accuracy (green line, A) still stabilizes close to 1.0, while the validation accuracy (blue line, A) fluctuates within the same range (0.65–0.85), displaying the same instability as in **Supplementary-Fig. 1**. Similarly, the training and validation loss curves in **Supplementary-Fig. 2** (B) follow a similar trend to those in **Supplementary-Fig. 1**, with low and stable training loss but some oscillations in validation loss.

These results underscore the relatively small size of the visible dataset, which comprises only 550 images. Despite employing data augmentation to expand the training set artificially, the underlying features and distributions of the dataset remain unchanged. This inherent limitation may hinder the model's ability to generalize effectively, as reflected in the fluctuations in validation accuracy and loss curves in both figures. Larger and more diverse datasets could help in achieving more stable and reliable validation performance, thereby improving generalization.

Supplementary-Fig. 3 and Supplementary-Fig. 4 shows the training and validation accuracy and loss curves for the infrared dataset from the VIRI Database, comparing the performance of the ResNet-18 model over 50 epochs, both with and without the CosineAnnealingLR scheduler.

In Supplementary-Fig. 3, where the CosineAnnealingLR scheduler is applied, the training accuracy (green line, A) rapidly increases and stabilizes close to 1.0, indicating that the model has effectively learned the features of the infrared training data. The validation accuracy (blue line, A) shows smoother and more consistent improvements compared to the results in Supplementary-Fig. 2, with less fluctuation and a higher peak. The loss curves in Supplementary-Fig. 3 (B) shows a similarly improved trend. The training loss (green line) remains low and stable throughout the epochs, while the validation loss (blue line) gradually decreases.

In contrast, Supplementary-Fig. 4 shows the performance of the same model without the CosineAnnealingLR scheduler. The training accuracy (green line, A) still increases rapidly and stabilizes close to 1.0, reflecting effective learning from the infrared training data. However, the validation accuracy (blue line, A) fluctuates significantly, peaking around 0.5. This indicates that the model struggles to generalize well on the infrared dataset, likely due to challenges in distinguishing emotional expressions from infrared features, as well as potential overfitting to the training data. The training loss in Supplementary-Fig. 4 (B) remains low and stable, showing effective minimization of errors in the training data. However, the validation loss (blue line) fluctuates without a clear downward trend, suggesting that while the model performs well on the training set, it struggles to minimize errors on the validation set. This suggests that the model may be overfitting or failing to capture key features needed for generalization on the infrared dataset.

The small size of the infrared dataset (comprising only 550 images) remains a significant factor in the observed instability of the validation performance. Despite data augmentation techniques, the inherent limitations in feature representation in infrared images could make it more difficult for the model to generalize effectively, as reflected in the fluctuating validation accuracy and loss curves.

**Hypothetical reasons for Instability:**

Here, we are providing possible explanations for the observed instability in the learning curves.

1. **Datasets:**
   1. **Dataset size:** The relatively small size of the datasets (550 images each for the visible and infrared datasets) significantly contributes to the observed instability in validation performance. Although data augmentation was applied to artificially expand the training set, this approach does not fundamentally alter the underlying feature distributions. For the visible dataset, the limited diversity may result in insufficient generalization, leading to fluctuating validation accuracy and loss curves.
   2. **Challenging modality (infrared):** Similarly, the infrared dataset faces additional challenges due to inherent limitations in feature representation from infrared images. These features are often less discriminative than those in visible images, making it harder for the model to learn robust patterns. Consequently, the combined visible and infrared dataset also inherits these shortcomings, further affecting stability and performance.

Larger and more diverse datasets, incorporating a broader range of features and samples, are essential to overcoming these limitations and achieving stable, reliable generalization in future work.

1. **Sharp Minima:**

The observed behavior in the learning curves can also be interpreted in the context of sharp minima during optimization, as illustrated in studies such as *LARS* (Layer-wise Adaptive Rate Scaling) [56]. The current model's optimization behavior indicates that it may be converging to sharp minima rather than flat minima, which exacerbates the instability in performance.

The average validation accuracy around the peak over a range of 10 epochs has been calculated to ensure a more robust representation of the model's performance**,** highlighting the inherent instability and feature limitations as shown in supplementary-Table 2. For the c**ombined visible and infrared datasets**, the average accuracy is **77.2%**, which is slightly lower compared to the visible dataset alone due to the observed fluctuations in the infrared dataset.
