# Supplementary figures and images for: Facial expression recognition using visible and IR by early fusion of deep learning with attention mechanism
Source: PeerJ Comput Sci. 2025 Mar 12;11:e2676. doi: 10.7717/peerj-cs.2676 (PMC11935750; doi:10.7717/peerj-cs.2676)

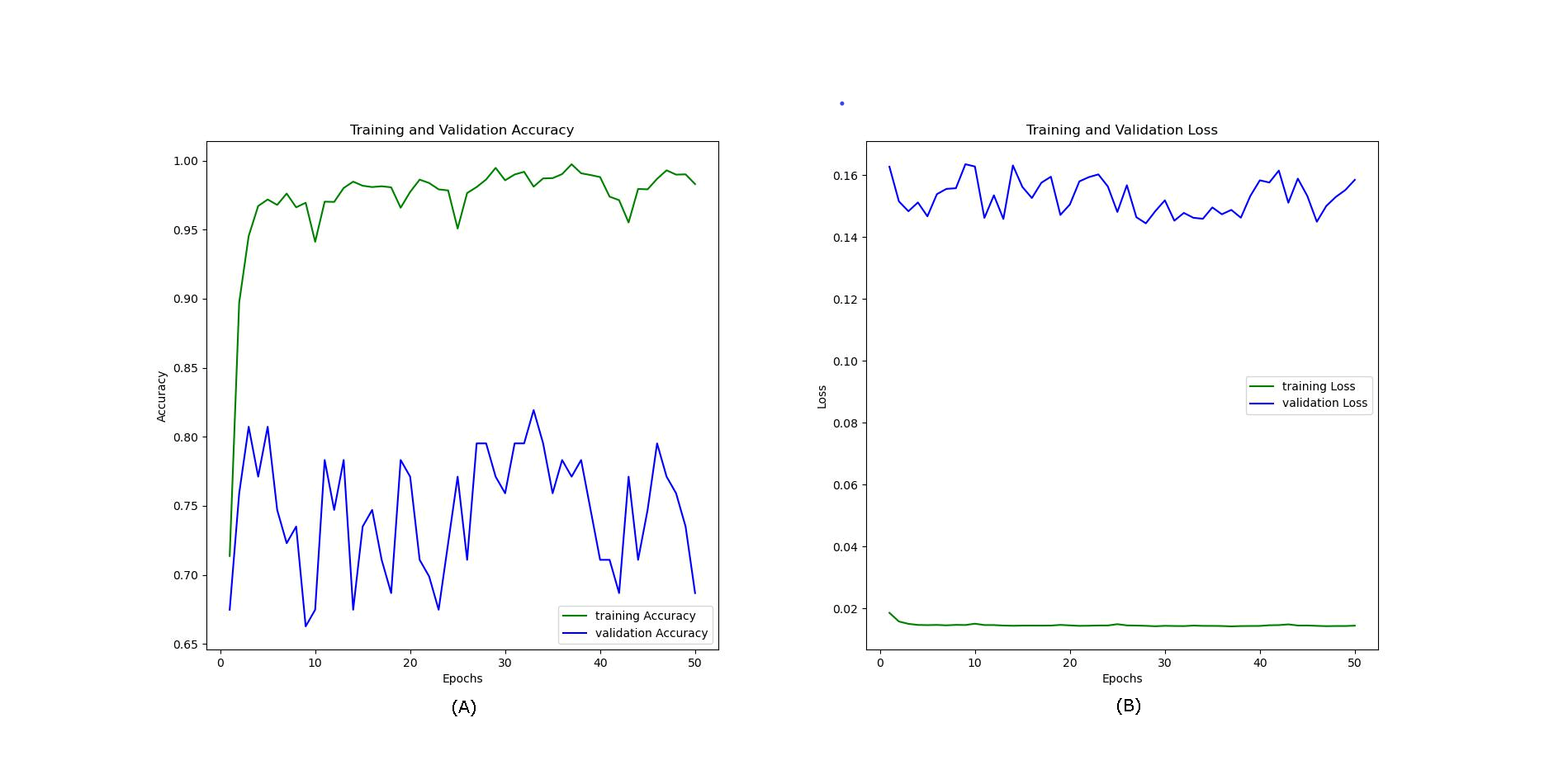

Supplement: Supplemental Information 3 [file peerj-cs-11-2676-s003.png]

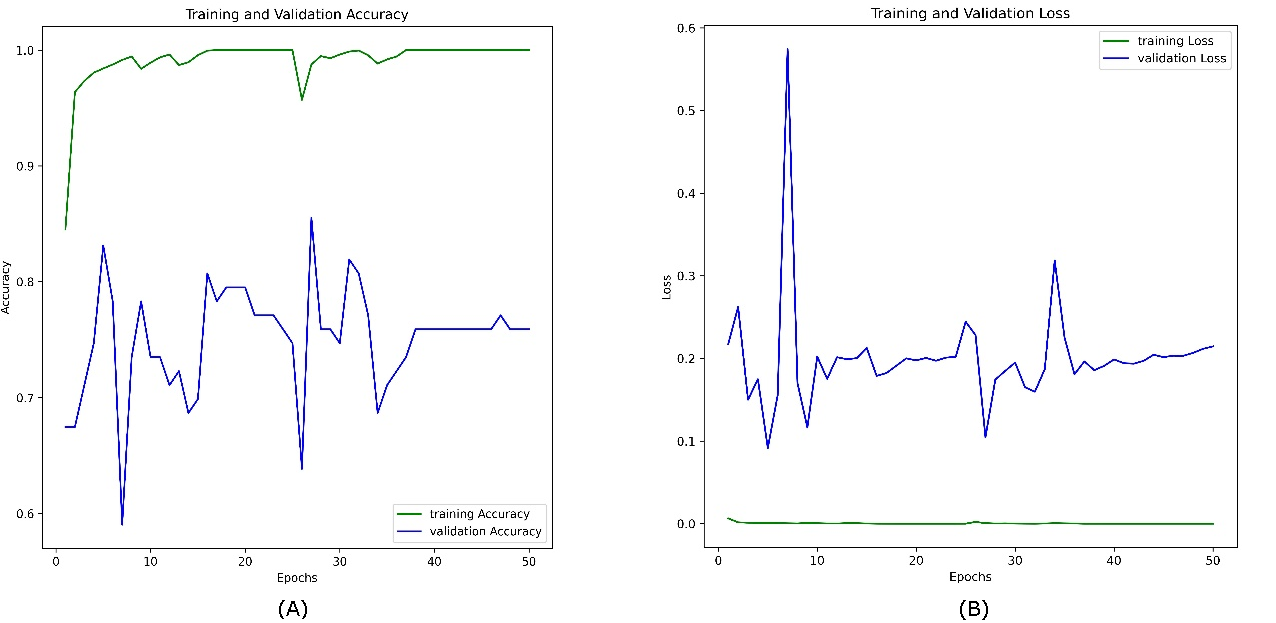

Supplement: Supplemental Information 4 [file peerj-cs-11-2676-s004.png]

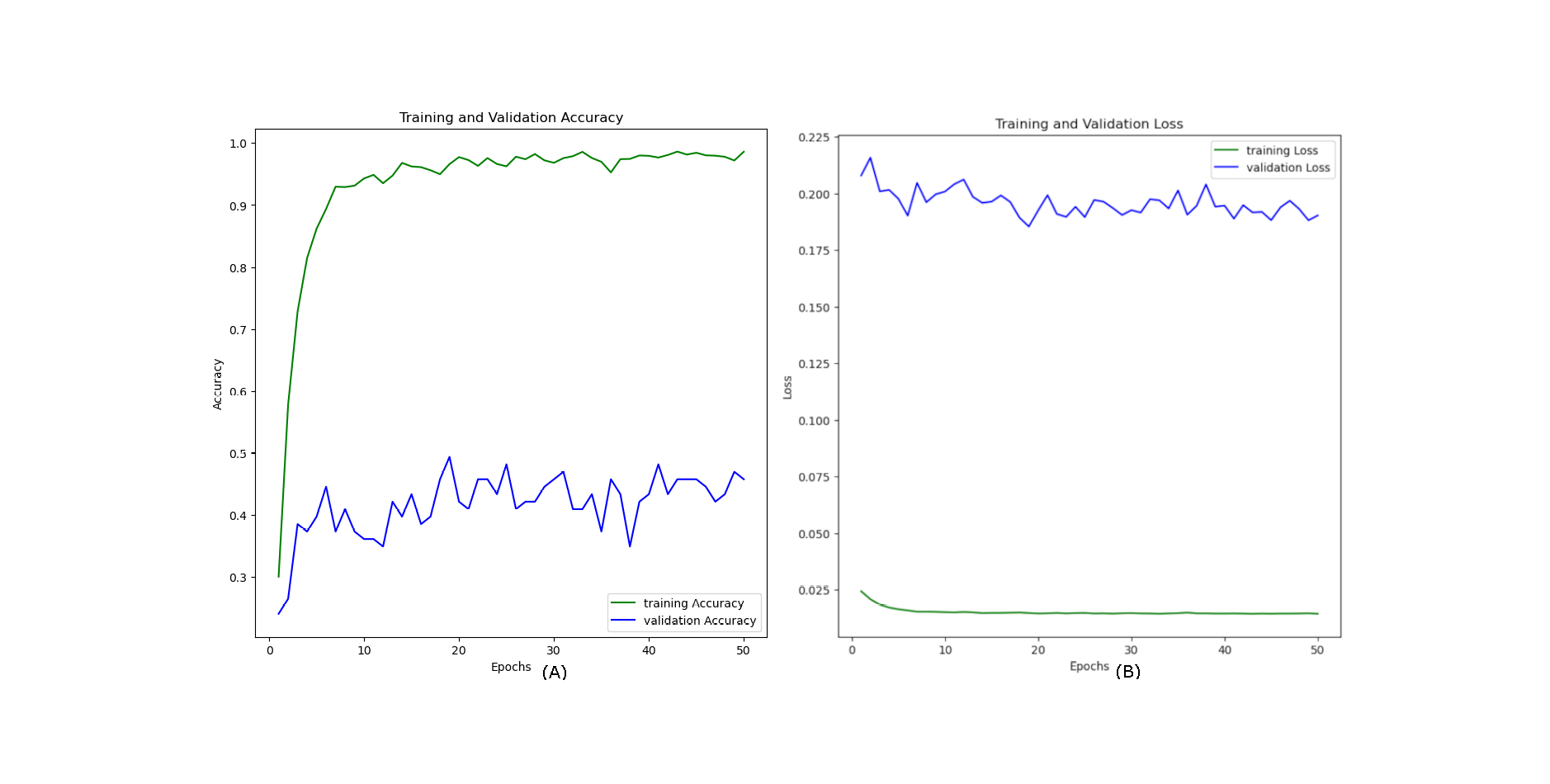

Supplement: Supplemental Information 5 [file peerj-cs-11-2676-s005.png]

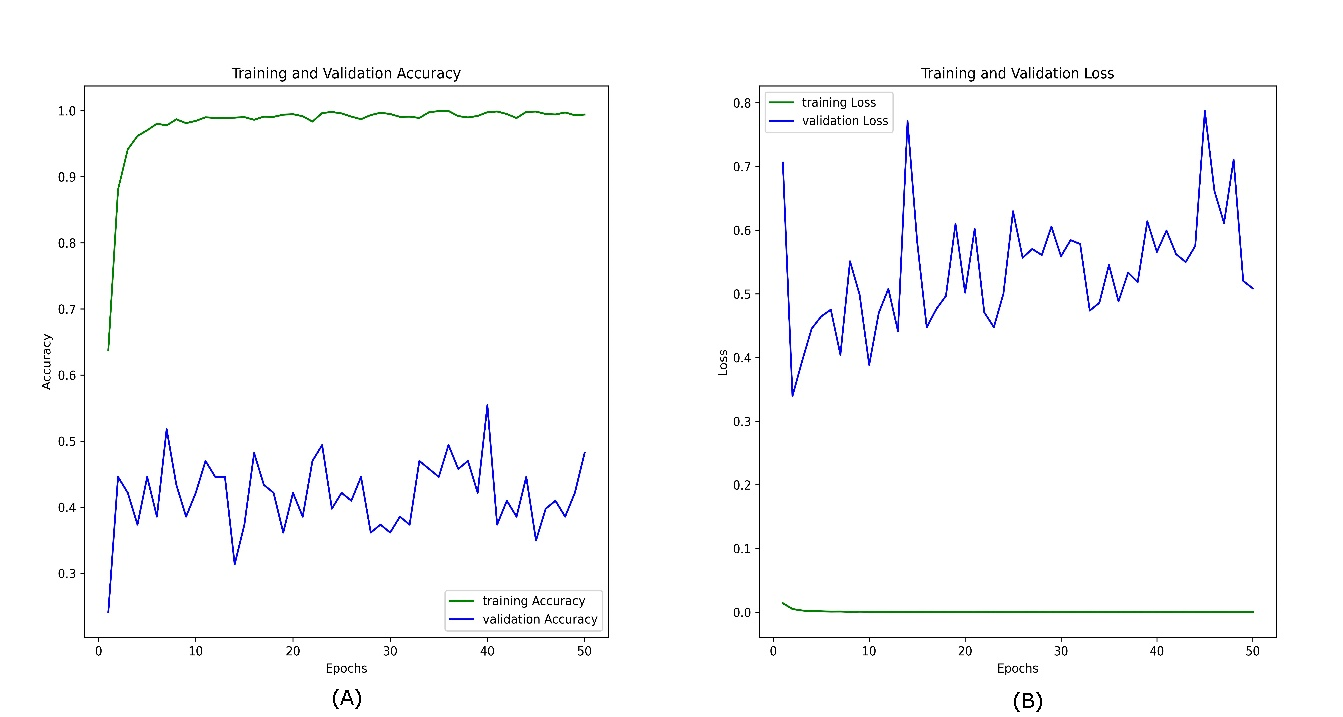

Supplement: Supplemental Information 6 [file peerj-cs-11-2676-s006.png]
