# Supplementary material for: Facial expression recognition using visible and IR by early fusion of deep learning with attention mechanism
Source: PeerJ Comput Sci. 2025 Mar 12;11:e2676. doi: 10.7717/peerj-cs.2676 (PMC11935750; doi:10.7717/peerj-cs.2676)
Supplement: Supplemental Information 7 [file peerj-cs-11-2676-s007.docx]

**Table 1:**

**Confusion matrix.**

| Predicted | | | |
| --- | --- | --- | --- |
|  | | **Positive** | **Negative** |
| Actual | **Positive** | True positive (TP) | False negative (FN) |
|  | **Negative** | False positive (FP) | True negative (TN) |
