# Supplementary material for: Facial expression recognition using visible and IR by early fusion of deep learning with attention mechanism
Source: PeerJ Comput Sci. 2025 Mar 12;11:e2676. doi: 10.7717/peerj-cs.2676 (PMC11935750; doi:10.7717/peerj-cs.2676)
Supplement: Supplemental Information 8 [file peerj-cs-11-2676-s008.docx]

| **visible** | **infrared** | **visible and infrared** |
| --- | --- | --- |
| 78.8% | 48.7% | 77.2% |
